# Efficacy and Safety of Switching from Prostaglandin Analog Therapy to Prostaglandin / Timolol Fixed Combination or Prostaglandin / Brimonidine Therapy

**DOI:** 10.2174/1874364101711010156

**Published:** 2017-06-30

**Authors:** Kenji Inoue, Mieko Masumoto, Kyoko Ishida, Goji Tomita

**Affiliations:** 1 Inouye Eye Hospital, 4-3 Kanda-surugadai, Chiyoda-ku Tokyo, 101-0062, Japan; 2 Department of Ophthalmology, Toho University Ohashi Medical Center, 2-17-6 Ohashi, Meguro-ku Tokyo, 153-8515, Japan

**Keywords:** Prostaglandin/timolol fixed combination, Brimonidine, Intraocular pressure, Prostaglandin analogues, Safety, Efficacy

## Abstract

**Background:**

To compare the safety and efficacy between switching to prostaglandin/timolol fixed combination eye drops (PG/timolol FCs) and adding brimonidine to PG analogue monotherapy.

**Methods:**

Eyes of 53 patients with primary open-angle glaucoma or ocular hypertension who were receiving PG analogue monotherapy were included. Participants were randomly divided into two treatment groups: one was prescribed PG/timolol FCs (switched group), and for the other, 0.1% brimonidine was added to the PG analogue (added group). Intraocular pressure (IOP), blood pressure, and pulse rate were measured after 1 and 3 months and compared to baseline values. Participants were also surveyed to determine if they had experienced systemic or topical adverse events at each study visit. IOP changes at 1 and 3 months were compared between groups.

**Results:**

Three months after changing medication, mean IOP was 14.6 ± 2.4 mmHg in the switched group and 13.7 ± 1.8 mmHg in the added group; both were significantly lower than the baseline values (switched group, 16.5 ± 2.7 mmHg; added group, 15.8 ± 2.3 mmHg; both P < 0.001). Neither the mean nor the percentage reductions in IOP were significantly different between groups at 1 and 3 months. In the added group, diastolic blood pressure was lower than that at 1 and 3 months, systolic blood was lower than that at 3 months (P < 0.01). The patients who had experienced systemic or topical adverse events were 53.8% in the added group and 40.7% in the the changed group, which was equivalent between groups (P =0.4142). Three patients (11.5%) in the added group, but none from the switched group, were excluded from analyses because of adverse events (not significant, P = 0.217).

**Conclusion:**

Switching from a PG analogue to PG/timolol FCs or to PG with brimonidine was equally safe (systemically and topically) and effective in reducing IOP. Thus, PG with brimonidine might be appropriate medication in patients who cannot use PG/timolol FCs due to repiratory or circulatory disease.

## INTRODUCTION

Glaucoma is often initially treated with a single medication that lowers intraocular pressure (IOP). Prostaglandin (PG) analogues are generally the first choice for glaucoma treatment because they are effective in reducing IOP, have few systemic adverse effects, and offer a convenient once-daily administration protocol. However, when PG analogues fail to sufficiently reduce IOP, a medication change or addition is needed. Switching patients from PG analogue monotherapy to PG/timolol fixed combination eye drops (PG/timolol FCs) is common [1] because of high patient compliance and the continued once-daily administration protocol. However, PG/timolol FCs, which contain a β-blocker, might not be used in patients with respiratory or circulatory disease. Fortunately, topical carbonic anhydrase inhibitors and α-2 stimulators might be both appropriate medication additions for patients who cannot use β-blockers. Brimonidine, an α2-adrenergic agonist, has been shown to have few systemic adverse effects, is effective in reducing IOP, and slows down the progression of visual field defects (*i.e.*, neuroprotective activity) [2]. Therefore, switching patients to PG/timolol FCs is favorable for patient compliance, while by adding brimonidine to PG analogue therapy, except greater IOP reduction, we might expect to achieve additional IOP-independent neuroprotection. In Japan, brimonidine 0.1% has only been approved for use as an adjunctive therapy since 2012.

A direct comparison between patients treated with PG analogue monotherapy who had switched to PG/timolol FCs with those who had brimonidine added to their medication regimen has not yet been performed in Japan. Here, we prospectively investigate the systemic and topical safety and efficacy of reducing IOP in Japanese patients by switching to PG/timolol FCs or by adding brimonidine to their glaucoma treatment regimen.

## MATERIALS AND METHODS

This study was conducted between April 2014 and October 2015 at the Inouye Eye Hospital (Tokyo, Japan). The protocol was approved by the hospital’s ethics committee and all participants provided written informed consent before any study procedure or examination was performed. All study conduct adhered to the tenets of the Declaration of Helsinki.

Study subjects had primary open-angle glaucoma (POAG) or ocular hypertension (OH) that was being treated with PG analogue which contains preservatives monotherapy administered once daily in the evening. All subjects showed insufficient IOP reduction after more than 3 months of latanoprost or travoprost treatment, necessitating a change in IOP-lowering medication. Subjects whose IOP reduction rate was < 20% using PG analogue monotherapy or visual field defects had increased were defined as IOP insufficient cases. Subjects with corneal disease whose IOP could not be precisely measured or who had undergone cataract surgery in the past 3 months were excluded from participation. In cases where both eyes qualified for study inclusion, the eye with the higher IOP was selected as the study eye. If both eyes had the same IOP, the right eye was selected as the study eye.

Participants were instructed to discontinue PG analogue in the study eye and to begin using study medication instead. Medications were not changed in the non-study eye. Subjects were randomly divided into two treatment groups using sealed envelopes. One treatment group began using PG/timolol FCs which contains preservatives monotherapy once daily in the evening (switched group) and one treatment group began using both a PG analogue (once daily in the evening) and 0.1% brimonidine which contains preservatives monotherapy (twice daily in the morning and evening; added group). In the switched group, 0.005% latanoprost was replaced by 0.005% latanoprost/0.5% timolol FCs and 0.004% travoprost was replaced by 0.004% travoprost/0.5% timolol FCs.

All subjects underwent ophthalmic examination, including measurement of IOP (Goldmann tonometry) before (baseline), 1 and 3 months after the use of the study medication and 30-2 SITA Standard Humphrey visual field testing before administration. Blood pressure (pulsimeter, UDEX super TYPE, Elquest Co., Ltd., Chiba, Japan) and pulse rate (pulsimeter, UDEX super TYPE, Elquest Co., Ltd., Chiba, Japan) were also measured at each time point. Topical adverse reactions were examined with slit-lamp microscopy and consultation. The IOP, blood pressure, and pulse rate were compared between baseline and 1 and 3 months within groups using paired *t* tests, and between groups at each time point using unpaired *t* tests. The reduction in IOP from baseline to 1 and 3 months, and the rate of reduction, were calculated and analyzed using the Wilcoxon signed rank test. Participants who dropped out of the study before the end of the 3-month observation period were also investigated. A P value < 0.05 was considered statistically significant.

## RESULTS

A total of 53 eyes of the 53 patients (21 men, 32 women) were included in this study. Mean subject age at baseline was 64.5 ± 12.4 years (range, 38–85 years). A total of 52 eyes had POAG, including 33 eyes with normal-tension glaucoma, and one eye had OH. Before beginning the study medication, 40 patients had been using latanoprost and 13 patients had been using travoprost for a period of 89 ± 42 months (range, 18-186 months).

Twenty-seven patients were assigned to the switched group and 26 patients were assigned to the added group Table (**1**). At baseline, there were no significant differences between the groups in gender, glaucoma type, pretreatment medications, number of medications used before the study, PG analogue administration period, baseline IOP, visual field mean deviation (MD), systolic blood pressure, diastolic blood pressure, and pulse rate. However, the subject age was significantly higher in the added group than in the switched group (*p* = 0.0034).

**Fig. (1):**
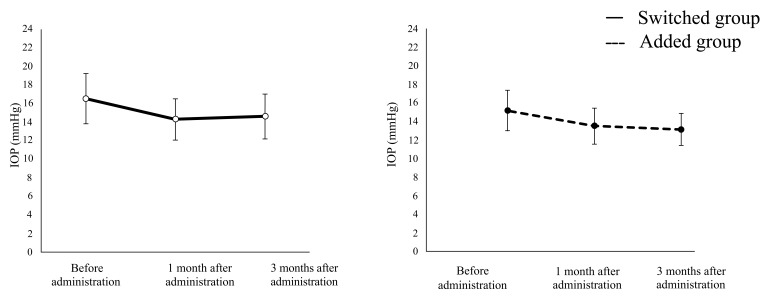
**Intraocular pressure following a change in glaucoma medication regimen.** The added group had brimonidine added to a prostaglandin analogue. The switched group switched from using a prostaglandin analogue to prostaglandin/timolol fixed combination eye drops. *p < 0.001 compared with pre-treatment values; **p < 0.0001 compared with pre-treatment values; IOP, intraocular pressure.

The mean IOP was significantly lower in the switched group after 1 (14.3 ± 2.2 mmHg) and 3 (14.6 ± 2.4 mmHg) months of study medication use compared with that at baseline (16.5 ± 2.7 mmHg, both p < 0.0001). Similar results were obtained in the added group (baseline IOP, 15.8 ± 2.3 mmHg; 1-month IOP, 14.1 ± 2.0 mmHg; 3-month IOP, 13.7 ± 1.8 mmHg; both P < 0.001; (Fig. **1**). The mean reduction in IOP after 1 month was 2.2 ± 2.1 mmHg in the switched group and 1.7 ± 2.5 mmHg in the added group; after 3 months, it was 2.0 ± 2.3 mmHg in the switched group and 2.1 ± 2.3 mmHg in the added group. These slight differences between the two groups were not statistically significant (1 month, P = 0.203; 3 months, P = 0.907). The IOP reduction rate at 1 month was 12.7 ± 11.3% in the switched group and 9.3 ± 14.1% in the added group (P = 0.185). The IOP reduction rate at 3 months was 11.2 ± 12.2% in the switched group and 12.1 ± 11.8% in the added group (P = 0.922). Again, these slight differences between groups were not statistically significant.

Neither systolic nor diastolic blood pressure measurements differed from baseline in the switched group after 1 and 3 months of study medication use Table (**2**). In the added group, however, systolic blood pressure at 3 months (*p* < 0.01) and diastolic blood pressure at 1 (P < 0.001) and 3 (P < 0.01) months were significantly lower than baseline. Pulse rate did not change from baseline at 1 or 3 months in either study group (1 month, P = 0.940; 3 months, P = 0.572). In the switched group, four patients (14.8%) developed systemic diseases, including hypertension (2 patients), hyperlipidemia (1 patient), and high blood pressure + hyperlipidemia (1 patient). In the added group, seven patients (26.9%) developed systemic diseases, including hypertension (2 patients), gout (1 patient), rheumatism (1 patient), prostatic hyperplasia (1 patient), diabetes (1 patient) and gastritis (1 patient) (Fisher's exact test, P = 0.3265).

Adverse events were experienced by 11 patients (40.7%) in the switched group, and 14 patients (53.8%) in the added group (Fisher’s exact test, P = 0.4112) Table (**3**). Three of the added group patients (11.5%), but none of the switched group patients, were excluded from analyses due to these systemic or topical adverse events. This slight difference in the number of excluded patients between groups was not significant (P = 0.217). In the added group, one subject experienced palpitations and chest pain, one had bradycardia and somnolentia, and one experienced ocular irritation.

## DISCUSSION

One study reported that adding brimonidine to PG analogues monotherapy or switching to a PG/β-blocker fixed combination are common practices that are favorable for patients [1]. However, the safety and efficacy of adding brimonidine to PG analogues monotherapy or switching to PG/β-blocker fixed combinations have not been investigated. Here, we examined the safety and efficacy in this study. Several studies have previously reported efficacy in IOP reduction when switching from PG analogues to PG/timolol FCs Table (**4**) [3-11]. When switching from latanoprost to latanoprost/timolol fixed combination eye drops, the IOP was reduced by 2.1-4.71 mmHg, or 11.2-19.5%, during the follow-up period from 2 to 6 months [3-7]. When switching from travoprost to travoprost/timolol fixed combination, IOP was reduced by 2.6-3.6 mmHg, or 13.6-28.5%, during the follow-up period from 3 to 24 months [8-11]. These IOP reductions are equivalent or lower than those previously reported [3-11]. However, baseline IOP (16.5 ± 2.7 mmHg) in our study subjects was lower (> 20 mmHg) than that of previous studies [3-11], which could explain this difference. The IOP reduction achieved by adding brimonidine to PG analogue therapy has also been examined previously Table (**5**) [12-20]. Brimonidine 0.1% has been used in Japan since 2012, but brimonidine 0.15% and 0.2% are used worldwide. In two reports of brimonidine 0.2% added to PG analogues, IOP was reduced by 2.3-5.9 mmHg and 13.4-32.2% during 1- to 2-month follow-up periods [12, 13]. In others, brimonidine 0.15% added to PG analogues delivered a 2.0–5.1 mmHg / 9–23% IOP reduction (6-week to 3-month follow-up) [14-16], while IOP reductions from a 0.1% brimonidine addition were 1.5-3.3 mmHg / 11.8-16.8% (4-week to 12-month follow-up) [17-22]. In agreement with prior studies [12-20], we found that mean IOP was 14.6 ± 2.4 mmHg over a 3-month follow-up period. In the current study, mean IOP was reduced by 2.0 ± 2.3 mmHg or 11.2 ± 12.2% over a 3-month (12-week) follow-up period.

Previous studies have also examined the usefulness of a change in medication to improve IOP management. One study examined the effect on IOP when patients with POAG and OH who had been using PG analogue and timolol concomitantly switched from timolol to 0.1% brimonidine [21]. In that study, IOP of 12 weeks after switching medications (14.0 ± 2.8 mmHg) was significantly lower than baseline (15.7 ± 2.7 mmHg).

Reis *et al.* [13] compared the efficacy between adding 0.2% brimonidine or timolol to PG analogue therapy. When brimonidine was added, the reductions and percentage change in mean IOP were 2.3 ± 1.8mmHg and 13.4 ± 9.1%, respectively. When timolol was added, reductions were even greater at 3.9 ± 1.8 mmHg and 20.2 ± 7.5%, respectively, both of which were significantly larger than for brimonidine [13].

Several comparative trials have examined the efficacy of brimonidine and timolol in reducing IOP [2, 22-25]. Krupin *et al.* [2] reported an equivalent IOP reduction efficacy between brimonidine and timolol, but Araie *et al*. [22] and Konstas *et al*. [23] reported that timolol use resulted in a greater IOP reduction. The peak IOP was reportedly the same in eyes treated with brimonidine or timolol; however, the trough IOP was lower in eyes treated with timolol than in those treated with brimonidine [24, 25]. In the current study, mean reductions and percentage changes in IOP were the same when PG analogue monotherapy was switched to either PG/timolol FCs or PG analogue with brimonidine therapy. When using a single PG analogue, the IOP reduction efficacy of timolol is greater than that of brimonidine [22, 23]. However, when additional eye drops were used with PG analogues, IOP reduction efficacy was equivalent between PG/timolol FCs and brimonidine added to PG analogues.

This study also examined the effect of study medications on blood pressure and pulse rate. Both brimonidine and timolol are known to mechanically lower blood pressure and pulse rate, and we found that brimonidine did lower these parameters. In the added group, similarly to previous studies [22], diastolic blood pressure was lower after brimonidine administration than before administration. However, the reduction was small and blood pressure remained within normal limits. Therefore, although the change was statistically significant, it was not clinically relevant.

Prior studies had a dropout rate of 1.5–6.5% because of adverse events when subjects switched from PG analogue monotherapy to PG/timolol FCs [6, 7, 10, 11]. Adverse effects included conjunctive hyperemia, keratitis, ocular irritation and itchiness, bradycardia, and blurred vision. However, none of our switched group subjects withdrew from the study because of adverse reactions. In other studies, up to 23.7% of patients dropped out because of adverse reactions after adding brimonidine to PG analogue therapy [13, 14, 16-20]. Adverse effects included increased blood pressure, reduced blood pressure, bradycardia, headache, tinnitus, and ocular itchiness. The added group in the current study had a withdrawal rate of 11.5% because of adverse reactions, which included palpitations, chest pain, bradycardia, somnolentia, and ocular irritation. The difference in adverse effect-related subject dropout rate between our added and switched groups was not statistically significant. However, three patients from the added group and none from the switched group withdrew from the study. The subject age was significantly higher in the added group than in the switched group, that may have influenced this result. Lastly, a 12-week study examined the effects of switching from timolol to brimonidine in patients who were using PG analogue. A total of 1.9% of patients dropped out due to adverse reactions, which included ocular itchiness and somnolentia [21]. The patients who had experienced systemic or topical adverse events were 53.8% in added group and 40.7% in the changed group, which was equivalent between groups (P =0.4142). The switched group used PG/timolol FCs once daily and the added group used both a PG analogues (once daily) and 0.1% brimonidine (twice daily), therefore we predicted that the added group experience more adverse events. The number of subjects and short-term evaluation may also have influenced the results of this study. The IOP was measured after few hours of administration thus conjunctive hyperemia was not evaluated accurately.

Our study had several limitations. First, the number of subjects was small. Second, we only followed subjects for 3 months after medication changes were made. Therefore, this study only evaluated short-term IOP reduction efficacy and safety. Future studies should feature longer follow-up periods and also examine the effect of these medications on the preservation of visual function over the long-term.

## CONCLUSION

In conclusion, this prospective study examined the safety and efficacy of two treatments in eyes of Japanese patients with POAG or OH. Both PG/timolol FCs and PG analogues plus brimonidine were equally safe and effective for lowering IOP. When PG analogues fail to sufficiently reduce Thus, PG with brimonidine might be appropriate medication in patients who cannot use PG/timolol FCs due to repiratory or circulatory disease. IOP, there was no difference between switching to PG/timolol FCs and adding brimonidine to PG analogue therapy. The patients’ adherence and systemic diseases should be considered.

## Figures and Tables

**Table 1:** Subject demographics and ocular characteristics.

	*Switched Group*	*Added Group*	*P Value*
n (patients)	27	26	---
Male: Female	13:14	8:18	0.264
Age (years)	59.7 ± 12.0 (42–85)	69.4 ± 10.9 (38–83)	0.0034
Systemic Diseases	4	7	0.3265
Glaucoma Type			0.217
POAG	12	7	
NTG	14	19	
OH	1	0	
Pretreatment medications			0.526
Latanoprost	19	21	
Travoprost	8	5	
PG treatment period (months)	88 ± 43 (32–168)	89 ± 41 (18–186)	0.838
IOP (mmHg)	16.5 ± 2.7 (11–24)	15.8 ± 2.3 (11–20)	0.278
Visual field MD (dB)	-9.69 ± 5.70 (-22.81–0.65)	-8.71 ± 5.84 (-17.99–1.18)	0.610
Systolic BP (mmHg)	129 ± 21(94–175)	135 ± 16(110–172)	0.314
Diastolic BP (mmHg)	77 ± 12(50–98)	76 ± 8(58–94)	0.676
Pulse rate	74 ± 10(56–97)	73 ± 9(51–86)	0.778

Data are presented as mean ± standard deviation. Data ranges are presented in parentheses.

POAG, primary open-angle glaucoma; NTG, normal-tension glaucoma; OH, ocular hypertension; PG, prostaglandin; IOP, intraocular pressure; MD, mean deviation; BP, blood pressure

**Table 2:** Blood Pressure and pulse rate in study subjects.

	*Baseline*	*1 month*	*3 months*
Systolic BP (mm Hg)			
Switched group	129 ± 21	127 ± 18	124 ± 18
Added group	135 ± 16	128 ± 19	124 ± 21*
Diastolic BP (mmHg)			
Switched group	77 ± 12	74 ± 11	76 ± 12
Added group	76 ± 8	68 ± 8**	71 ± 10*
Pulse rate (beats/minute)			
Switched group	74 ± 10	74 ± 10	73 ± 9
Added group	73 ± 9	73 ± 9	75 ± 11

One- and 3-month values compared to baseline values using paired t-tests, *p < 0.05, **p < 0.001.

BP, blood pressure

**Table 3:** Adverse events after administration of study medication.

Onset date	Adverse events	Medication status following adverse event(s)		
Switched group	1 month	1 case	Ocular stimulation	Continuation
	1 month	1 case	Eyelid pigmentation	Continuation
	1 month	1 case	Superficial punctate keratopathy	Continuation
	1 month	1 case	Impaired vision	Continuation
	3 months	2 cases	Itchiness	Continuation
	3 months	4 cases	Impaired vision	Continuation
	3 months	1 case	Eye-smarting	Continuation
Added group	1 month	1 case	Palpitation, Chest pain	Discontinuation
	1 month	1 case	Bradycardia, Somnolentia	Discontinuation
	1 month	1 case	Oppressive feeling	Continuation
	1 month	1 case	Eye strain	Continuation
	1 month	1 case	Dizziness	Continuation
	1 month	1 case	Discomfort	Continuation
	1 month	3 cases	Impaired vision	Continuation
	1 month	1 case	Hyperemia	Continuation
	3 months	1 case	Ocular irritation	Discontinuation
	3 months	1 case	Discomfort	Continuation
	3 months	1 case	Blepharospasm	Continuation
	3 months	1 case	Itchiness	Continuation

**Table 4:** Intraocular pressure reduction efficacy in subjects switched from prostaglandin analogue monotherapy to prostaglandin/timolol fixed combination eye drops.

	*n (patients)*	*Prostaglandin/timolol FCs*	*Pre-treatment IOP (mmHg)*	*IOP reduction (mmHg)*	*IOP reduction rate (%)*
Latanoprost→Latanoprost/timolol fixed combination					
Hamacher T. Br J Ophthalmol. 2004 [3]	69	2 months	20.8 ± 3.4	3.1 ± 3.6	14.9
Dunker S. Adv Ther. 2007 [4]	51	6 months	20.7 ± 3.6	2.9 ± 2.8	14.0
Polo V. Ann Ophthalmol. 2008 [5]	33	3 months	20.38 ± 5.33	4.71	19.5
Kitazawa Y. Rinsho Ganka. 2009 [6]	144	8 weeks	19.62 ± 2.60	2.59 ± 2.40	13.2
Inoue K. Clin Ophthalmol. 2012 [7]	31	6 months	17.3 ± 2.7	2.1 ± 2.3	11.2 ± 11.8
Travoprost→Travoprost/timolol fixed combination					
Mandic Z. Methods Find Exp Clin Pharmacol. 2010 [8]	45	3 months	22	4.4 ± 2.8	20
Pfeiffer N. Clin Ophthalmol. 2010 [9]	45	12 weeks	22.1 ± 2.7	6.3 ± 2.5	28.5
Costa VP. Clin Ophthalmol. 2012 [10]	43	12 weeks	20.5 ± 2.1	3.9	19
Muraki T. Rinsho Ganka. 2015 [11]	34	2 years	16.9 ± 3.3	2.6 ± 2.9	13.6 ± 14.9
This study: PG analogue → PG/timolol fixed combination					
	27	12 weeks	16.5 ± 2.7	2.0 ± 2.3	11.2 ± 12.2

Data presented as mean ± standard deviation.

IOP, intraocular pressure; PG, prostaglandin

**Table 5:** Intraocular pressure reduction after adding brimonidine to the medication regimen of patients already using a prostaglandin analogue.

	*n (patients)*	*Drug*	*Treatment period*	*IOP before brimonidine (mmHg)*	*IOP reduction width (mmHg)*	*IOP reduction rate (%)*
Brimonidine 0.2%						
Lee DA. J Glaucoma. 2001 [12]	16	Latanoprost	2 months	18.3	5.89	32.2
Reis R. Clin Ther. 2006 [13]	16	Travoprost	4 weeks	17.0 ±3.1	2.3 ± 1.8	13.4 ± 9.1
Brimonidine 0.15%						
Konstas AGP. Ophthalmology. 2005 [14]	29	Latanoprost	6 weeks	19.0 ± 1.7	2.2 ± 1.5	11.6
Mundorf T. Adv Ther. 2007 [15]	43	Latanoprost	2 months	21.9	Peak 5.1 Trough 2.0	23 9
Feldman RM. Ophthalmology. 2007 [16]	79	Travoprost	3 months	21.7 ± 0.33	2.1 ± 0.27	9.7
Brimonidine 0.1%						
Day DG. Curr Med Res Opin. 2008 [17]	20	Latanoprost	3 months	19.6 ± 2.94	3.3 ± 2.82	16.8
Araie M. Atarasii Ganka. 2012 [18]	59	PG analogues	52 weeks	18.7	2.7	14.4
Yamamoto C. Atarasii Ganka. 2014 [19]	24	PG analogues	3 months	18.0 ± 2.7	2.1 ± 2.2	11.8 ± 11.4
Hayashi Y. Rinsho Ganka. 2015 [20]	17	PG analogues	12 months	11.5 ± 1.5	1.5 ± 1.3	13.3 ± 10.9
This study (latanoprost or travoprost)						
	26	PG analogues	12 weeks	15.8 ± 2.3	2.1 ± 2.3	12.1 ± 11.8

Data presented as mean ± standard deviation.

IOP, intraocular pressure; PG, prostaglandin.
